# Efficacy and safety of medical cannabinoids in children: a systematic review and meta-analysis

**DOI:** 10.1038/s41598-021-02770-6

**Published:** 2021-12-06

**Authors:** Nir Treves, Noa Mor, Karel Allegaert, Hely Bassalov, Matitiahu Berkovitch, Orit E. Stolar, Ilan Matok

**Affiliations:** 1 Division of Clinical Pharmacy, School of Pharmacy, Faculty of Medicine, The Hebrew University of Jerusalem, Jerusalem, Israel; 2 Department of Development and Regeneration, KU Leuven, Leuven, Belgium; 3 Department of Pharmaceutical and Pharmacological Sciences, KU Leuven, Leuven, Belgium; 4 Department of Hospital Pharmacy, Erasmus MC University Medical Center, Rotterdam, The Netherlands; 5 Clinical Pharmacology Unit, Shamir Medical Center (Assaf Harofeh), Zerifin, Sackler Faculty of Medicine, Tel-Aviv University, Tel Aviv, Israel; 6 The Autism Center, Alut, Shamir Medical Center (Assaf Harofeh), Zerifin, Israel; 7 Department of Clinical Pharmacy, Institute for Drug Research, School of Pharmacy and the David R. Bloom Center of Pharmacy, Faculty of Medicine, The Hebrew University of Jerusalem, Jerusalem, Israel

**Keywords:** Diseases, Health care, Medical research, Neurology

## Abstract

Despite the increased use of medical cannabinoids, the efficacy and safety of the treatment among children remain uncertain. The objective was to study the efficacy and safety of medical cannabinoids in children. The search included studies through 11-May-2020. Selection criteria included studies evaluating efficacy and safety outcomes of medical cannabinoids (tetrahydrocannabinol, cannabidiol and other cannabis derivatives) versus control in children, independently assessed by two reviewers. Eight studies were included, all of which are randomized controlled trials. Cannabidiol is associated with 50% reduction in seizures rate (Relative Risk (RR) = 1.69, 95% CI [1.20–2.36]) and caregiver global impression of change (Median Estimated difference = (− 1), 95%CI [− 1.39–(− 0.60)]) in Dravet syndrome, compared to placebo. While cannabidiol was associated with a reduction in reported seizure events (RR = 0.59, 95% CI [0.36–0.97]), no association was found in products contained also tetrahydrocannabinol (RR = 1.35, 95% CI [0.46–4.03]). Higher dose of cannabidiol was associated with decreased appetite (RR = 2.40, 95% CI [1.39–4.15]). A qualitative assessment suggests that medical cannabinoids might be associated with adverse mental events. In conclusion, cannabidiol is associated with clinical improvement in Dravet syndrome. However, cannabidiol is also associated with decreased appetite. Adverse mental events were reported as well, however, more research should be performed to assess well this outcome.

## Introduction

The popularity of medical cannabinoids (MCs) treatment is growing, as more countries enable MCs treatment for various indications in adults^1^. This trend is also observed in the pediatric population, where MCs are authorized in children for refractory epilepsy, especially in Dravet syndrome and Lennox-Gastaut syndrome^2^. Dravet syndrome is a rare presentation of intractable epileptic encephalopathies associated with pleomorphic seizure activity, usually before the one year of age, while Lennox-Gastaut syndrome is characterized by multiple drug-resistant seizure with a unique EEG pattern. The infants suffer from cognitive decline, motor, and behavioral abnormalities^3,4^. Other presentations of epilepsy and additional indications, such as autism, and inflammatory bowel disease (IBD) have been reported recently^5–7^ The non-psychoactive substance cannabidiol (CBD) is used for these indications, whereas chemotherapy-induced nausea and vomiting (CINV) are treated with the psychoactive D-9-Tetrahydrocannabinol (THC) and its analogues^8–10^.

However, despite the increased use of MCs in children, there is limited data regarding their safety and efficacy in this population for those different indications^11^. Pharmacotherapy in children has other safety-benefit profiles for most drugs than those seen in adults due to differences in physical characteristics, developmental aspects, and different prognosis and disease manifestation^12,13^. In adults, cannabinoids-related adverse events were previously associated with physiological reactions, including hyperthermia, rhabdomyolysis, increased appetite, and hypoglycemia, mostly in exposure to THC^14–16^. Furthermore, exposure to cannabis for recreational use has been previously associated with cognitive and behavioral outcomes, namely induction of psychosis and schizophrenia, cognitive compromise in adolescents, and depression^17–20^. Reported symptoms of unintentional cannabis ingestion in children include tachycardia and mydriasis but are characterized mainly by neurologic abnormalities such as lethargy, ataxia, and prolonged coma^21–24^. Evidence suggests that in-utero exposure to cannabis may pose neurodevelopmental outcomes, such as autism and a possible association between marijuana detected in breast milk and decreased motor development in infants^25,26^.

A systematic review published in 2017 aimed to identify available data about the therapeutic effect of cannabinoids treatment in children and adolescents. The authors hereby found that evidence was limited and mainly was considered low quality, and concluded that additional research is needed to evaluate the cannabis risk–benefit balance^27^. Since 2017, several clinical trials and observational studies have examined the efficacy of MCs in the pediatric population for a few indications, most of them focused on CBD efficacy in Dravet syndrome^28–30^.

Previous meta-analyses reported that CBD in epileptic adults and children in one cohort decreased the risk for seizures compared to placebo. However, these meta-analyses also reported the elevated risk for treatment withdrawals and adverse events such as somnolence, decreased appetite, diarrhea, and increased serum aminotransferases^31,32^.

This study aimed to assess the safety and efficacy of MCs in children using systematic review, meta-analysis, and advanced meta-analysis methods.

## Methods

### Search strategy

Systematic review and meta-analysis were performed to examine the efficacy and safety of MCs treatment among children. The systematic review was conducted according to the framework guidelines of Preferred Reporting Items for Systematic Reviews and Meta-Analysis (supplementary Table S1)^33^. The search included published and unpublished studies through 11-May-2020. The systematic review included MEDLINE, EMBASE, and ‘clinicaltrials.gov’ databases according to pre-selected keywords.

The protocol included a search strategy for keywords such as “medical cannabis”, “cannabis”, “medical marijuana”, “THC”, “CBD”, “dronabinol”, “nabiximols”, “adolescents”, “child”, “efficacy”, “safety”, and “adverse reactions”. Randomized control trials (RCTs) and observational studies were included in the search with no language nor date restrictions. The study protocol was registered in the International Prospective Register of Systematic Reviews (PROSPERO) and was updated as the screening progressed (CRD-42019132383) (supplementary Method). Considering the study design, no ethical review board assessment was required.

### Study selection

Two independent investigators (Noa Mor and Nir Treves) screened publications with Rayyan QCRI, a web and mobile app for systematic reviews^34^. The publications were screened based on their relevance to the selection criteria. The selection criteria included studies that evaluated the efficacy and safety outcomes of MCs treatment compared to placebo or other pharmacotherapy for any medical purpose among the pediatric population (≤ 18 years old). The screening was focused on studies that examined the efficacy and safety outcomes of active ingredients in cannabis, such as THC, CBD, and additional cannabinoids such as Nabilone. Due to scarce data and since the reported outcomes were expected to vary, no specific efficacy or safety outcomes were defined before data collection to facilitate a comprehensive evaluation of the available studies in this area. All potentially eligible studies were considered regardless of study design.

At initial screening, the studies were assessed independently for potential inclusion by title by the two investigators. A reason for exclusion for every individual study was documented. Disagreements were resolved by discussion. Following an initial screening, the included studies were reviewed once for evaluation based on the abstract, with disagreements resolved by discussion.

Following the abstract screening, the full text of eligible publications was examined, and a final decision for inclusion was made. At this point, studies with no comparison group or when exposure was not MCs were excluded from the meta-analysis. For eligible publications with missing data, the corresponding author was contacted to access additional data. If these attempts failed, the information as available was utilized to include the data when applicable. In addition, citations in the selected articles were reviewed independently by the two investigators (Noa Mor and Nir Treves) for identifying additional eligible articles.

### Data extraction

Comprehensive data extraction from each article was conducted by the two investigators independently according to an extraction form created and agreed upon in advance (variables specified in Supplementary Method). Study quality was assessed for the risk of bias using the tool Risk of Bias in Non-Randomized Studies of Interventions for observational studies (Robin-I)^35^ and Revised Cochrane risk-of-bias tool for randomized trials (RoB-2)^36^. This assessment was also conducted separately, and differences were resolved by discussion.

Since only a limited number of studies, each assessing several outcomes was expected, the precise outcomes were defined during data extraction process, for avoiding any important trends and implications. Adverse events with common pharmacological mechanisms or standard system organ class were grouped in the same analysis. The following outcomes were examined:

- The outcomes of 50% reduction in seizure rate from baseline; Caregiver Global Impression of Change (CGIC), a subjective assessment of the caregiver’s view of the patient's global functioning, measured in an ordinal scale^37^; and reported seizures events were measured as epilepsy efficacy outcomes in CBD treatment vs. placebo.
- The mean difference of vomits and retching events in nabilone (synthetic THC) vs. control was evaluated as an efficacy outcome in CINV.
- Number of patients who suffered from serious adverse events (SAEs), as described in the studies’ articles, clinicaltrials.gov (supplementary Method) or listed in the critical medical events list of the European Medical Agency^38^.
- Decreased appetite was defined as a binary outcome.
- Signs and symptoms of gastrointestinal hyperactivity were grouped, including events of diarrhea, upset stomach, nausea, retching and vomit. Due to the nature of reports in the included studies, more than one event can occur in one participant.
- Adverse mental events. Since various adverse mental events can be interpreted differently by the patients, caregivers and the researchers, a united outcome of adverse mental events was measured. Due to the nature of reports in the included studies, more than one event can occur in one participant. This outcome included the following events: fatigue, somnolence, unresponsive to stimuli, vagueness, lightheadedness, excitability, irritability, hysteria crying, aggression, abnormal behavior, psychomotor hyperactivity, hallucinations, elevation of mood, mood changes, euphoria. The full list of adverse mental event is detailed in the supplementary Methods.
- The outcome of infections included the infective events from pathogenic agents reported in the studies (supplementary Method).
- Pyrexia, as reported in the included studies.

### Statistical analysis

The possible impact of variations in study design and different formulations was addressed by measuring heterogeneity and utilizing random-effects models. I^2^ was calculated as the degree of heterogeneity observed in the analysis. The *p*-value < 0.1 was considered statistically significant. When heterogeneity was observed, a further investigation was performed in subgroup analyses, divided by products composite: CBD products, CBD and THC mixed products, and THC\THC-like products. As meta-regression was not applicable due to small number of studies and available dosages a network meta-analysis (NMA) was conducted to estimate the dosage impact on the measured results in a head-to-head method and heterogeneity.

Pooled data analysis was performed with R programming language with R-studio platform, using “meta”, “netmeta”, “metamedian”, “metafor”^39–42^. The pooled risk-ratios was calculated and 95% confidence of intervals (CI) to summarize the results for the dichotomous outcomes assessed for children treated with MCs versus children who were treated with placebo or control treatment. Sidik-Jonkman method was used, which provides conservative results and wider confidence intervals (Supplementary Method). Trial sequential analysis (TSA) was utilized to quantify the required sample size to determine the effect of outcomes while adjusting the threshold for statistical significance, with the Sidik-Jonkman model and O’brein Fleming boundaries function, using the trial sequential analysis program v.0.9-beta (http://www.ctu.dk/tsa/downloads.aspx)^43^. Two-sided trial sequential analysis was conducted to maintain a risk of 5% for type I error and a power of 80%, using the retrieved data from included studies and to estimate the required information size (IS). The information size is the total number of subjects and events that are necessary to detect or reject an assumed intervention effect in a meta-analysis^44^.

For pooling data of additional efficacy measured by an ordinal variable, the quantile estimation method was used. Analysis of results and mean difference calculation were extracted solely from studies with a crossover design and was performed by Comprehensive Meta-Analysis (CMA) software to consider the premises of this unique study design during pooled data calculations^45^. Outcomes evaluated by only one of the included studies are not shown in the meta-analysis. The outcome of adverse mental events was not analyzed as a pooled analysis since the number of events were higher than number of the participants in some of the comparison groups. Therefore, a summary table of adverse mental events is presented with Conditional maximum likelihood estimate of Rate Ratio and Fischer exact test^46^. Since less than ten studies were included, funnel plots were not used to assess publication bias^47^.

## Results

Out of 9133 results, ten articles met the inclusion/exclusion criteria. Three studies were excluded because of missing data, despite trying to contact the authors, and one additional study was found in the references of eligible articles (Supplementary Fig. S1). Eventually, eight remaining studies were RCTs conducted with MCs indicated for epilepsy (CBD extract), severe behavioral problems (CBD extract), CINV (nabilone) and spasticity (nabiximols, a 1:1 formulation of THC and CBD), and autism spectrum disorder (ASD) (whole-plant cannabis extract, purified CBD, and THC, both contained CBD and THC in ratio 20:1 respectively), with a total of 642 patients^28,48–54^. Table1 presents the included studies in the meta-analysis. According to quality assessment (Supplementary Fig. 2), five of the included studies posed a low risk of bias, one posed some concerns for bias, while the remaining two were judged to be at high risk of bias. Supplementary Table S2 presents a summary of findings of all meta-analyses conducted.

**Table 1:** Characteristics of studies included in analysis.

Authors and year of publication	Study type	Number of participants	Range of ages (years)	Route of administ-ration	Intervention and control	Indication	Follow up period	Funding
Dalzell et al.^49^	RCT. double blind, crossover	23^a^	0.8–17.0	Oral capsules or white powder	Nabilone vs. domperidone	Chemotherapy-Induced Emesis	Two cycles of chemotherapy^b^	Eli Lilly
Chan et al.^48^	RCT, double-blind, crossover	36^a^	3.5–17.8	Oral capsules	Nabilone vs. prochlorperazine	Chemotherapy-Induced Emesis	Two cycles of chemotherapy^b^	Eli Lilly
Devinsky et al.^28^	RCT, double-blind	120	2.3–18.4	Oral solution	CBD 20 mg/kg/d vs. Placebo	Dravet syndrome	14-week treatment period, a 10-day taper period, and a 4-week safety follow-up period	GW Pharmaceuticals
Devinsky et al.^30^	RCT, double-blind	34	4.0–10.9	Oral solution	CBD 5,10,20 mg/kg/d vs. Placebo	Dravet syndrome	3-week treatment, 10-day taper, and 4-week safety follow-up periods	GW Pharmaceuticals
Miller et al.^50^	RCT, double-blind	199	2.0–18.0	Oral solution	CBD dose 10,20 mg/kg/d vs. Placebo	Dravet syndrome	2-week titration period followed by a 12-week maintenance period	GW Pharmaceuticals
Fairhurst et al.^51^	RCT, double-blind	72	8.0–18.0	Spray	max 12 actuations of Nabiximols spray^c^ vs. Placebo	Spasticity due to cerebral palsy or traumatic, non‐progressive CNS injury	Patients titrated over a period of 9 weeks followed by 3 weeks of maintenance to a total of 12 weeks	GW Pharmaceuticals
Efron et al.^52^	RCT, double-blind	8	8.0–16.0	Oral solution	CBD 20 mg/kg/d vs. Placebo	Severe behavioral problems with intellectual disability	9-days up-titration, followed by 8 weeks of maintenance and 9 days down-titration	Internal grant (not commercial sponsor)
Aran et al^53^	RCT, double-blind, crossover	150	5–21	Oral solution	whole-plant cannabis extract, purified CBD and THC vs. Placebo^d^	Autism spectrum disorder	12-weeks, followed by a 4-week washout and cross-over for another 12 weeks	BOL Pharma and the National Institute for Psychobiology in Israel

^a^These two RCT’s were rated low in quality assessment in RoB-2, as detailed in Supplementary Fig. 2.

^b^These two RCT’s did not specify what was follow up period after treatment.

^c^Every actuation of spray contains 2.7 mg THC, 2.5 mg CBD. The study reported that the mean dosage was 15.7 ± 4.4 mg THC and 14.6 ± 4.0 mg CBD.

^d^Both extracts contained CBD and THC in ratio 20:1 respectively. The study reported that the mean dosage was 5.8 mg/kg a day.

**Figure 1:**
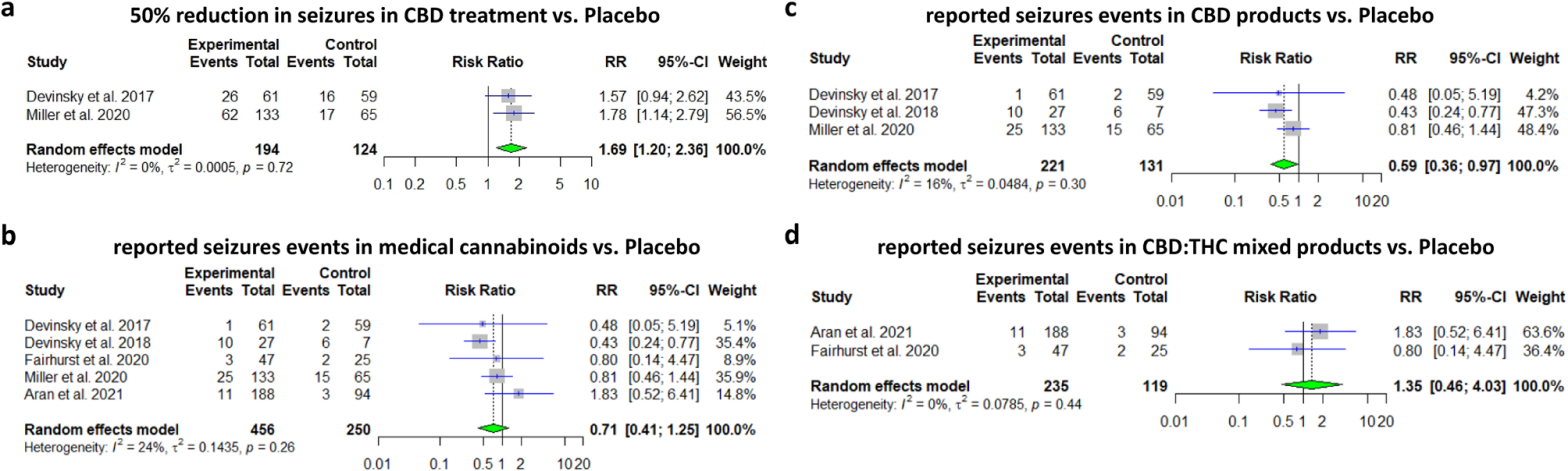
Efficacy in convulsions reduction during medical cannabinoids treatment. (**a**) Meta-analysis: 50% reduction in seizures in CBD treatment vs. Placebo (*p*-value = 0.002). (**b**) Meta-analysis: reported seizures events in medical cannabinoids vs. Placebo (*p*-value = 0.24). (**c**) Meta-analysis: reported seizures events in CBD products vs. Placebo (*p*-value = 0.04). (**d**) Meta-analysis: reported seizures events in CBD: THC mixed products vs. Placebo (*p*-value = 0.58).

**Figure 2:**
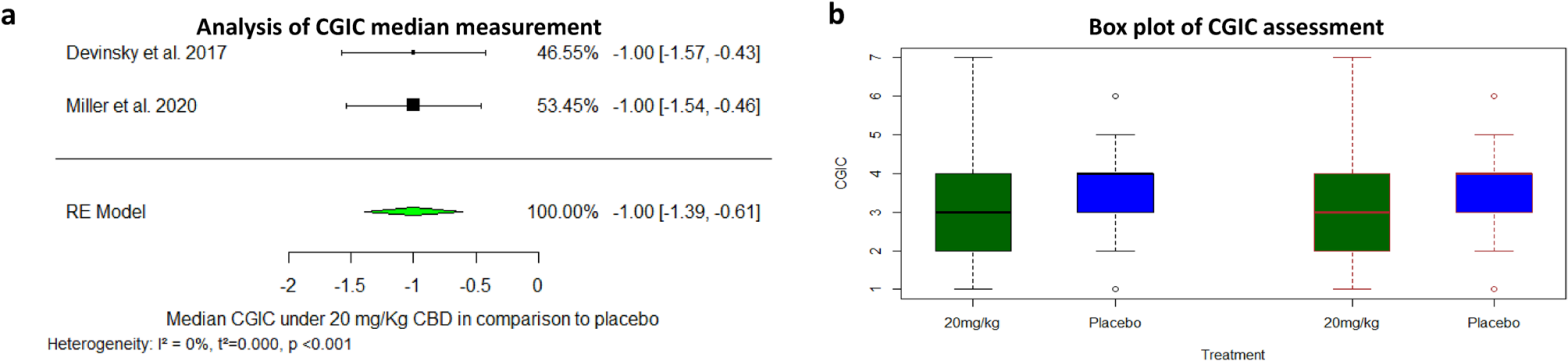
(**a**) Analysis of CGIC median measurement. Median estimated difference = (− 1), CI [− 11.39, − 0.60] (*p*-value < 0.001). (**b**) Box plot of CGIC assessment: Devinsky 2017 (left) and Miller 2020 (right), Dots mark outliers.

Five studies have reported on outcomes related to seizures control: Three of them assessed CBD treatment in Dravet syndrome, whereas the interventions in the remaining two studies included mixed CBD-THC extracts assessing their efficacy for the treatment in spasticity and ASD (and yet reported on seizure events within the studies). The pooled relative risk showed that CBD likely results in a 50% reduction in seizure rate from baseline (RR = 1.69, 95% CI [1.20–2.36], *p*-value = 0.002, I^2^ = 0%). A pooled analysis based on five included studies showed that CBD-containing products were not associated with decreased seizures events vs. placebo (RR = 0.71, 95% CI [0.41–1.24], *p*-value = 0.23, I^2^ = 23%). A subgroup analysis focusing in pure CBD products suggests that these products probably decrease reported seizure events vs. placebo (RR = 0.59, 95%CI[0.36 –0.97], *p*-value = 0.03, I^2^ = 16%), while mixed THC:CBD products (nabiximols used in the study conducted by Fairhurst et al., and 20:1 CBD:THC extraction used in the study conducted by Aran et al.) are not associated with this decrease (RR = 1.35, 95% CI [0.46–4.03], *p*-value = 0.59, I^2^ = 0%). The pooled analysis showed a reduction in CGIC Median estimated (Median estimated difference = (− 1), 95% CI [− 1.39–(− 0.60)], *p*-value < 0.001, respectively) (Figs. 1a–d, 2a,b).

Two small-size crossover RCTs have examined the effect of nabilone as antiemetic in comparison to conventional treatment (prochlorperazine or domperidone) in children treated with chemotherapy, including a total of 48 participants. The evidence suggests nabilone results in a reduction in the number of reported events of vomits and retching (Difference in means: − 11.517, 95% CI [− 17.908–(− 5.127)], *p*-value < 0.001, I^2^ = 0%) (Supplementary Fig. 3, albeit both studies’ quality was assessed as poor (Supplementary Fig. 2). The outcome of nausea severity was not pooled since it was only available in one of the two studies, whereas the outcome of the patient’s preference did not fit any meta-analytic calculation.

**Figure 3:**
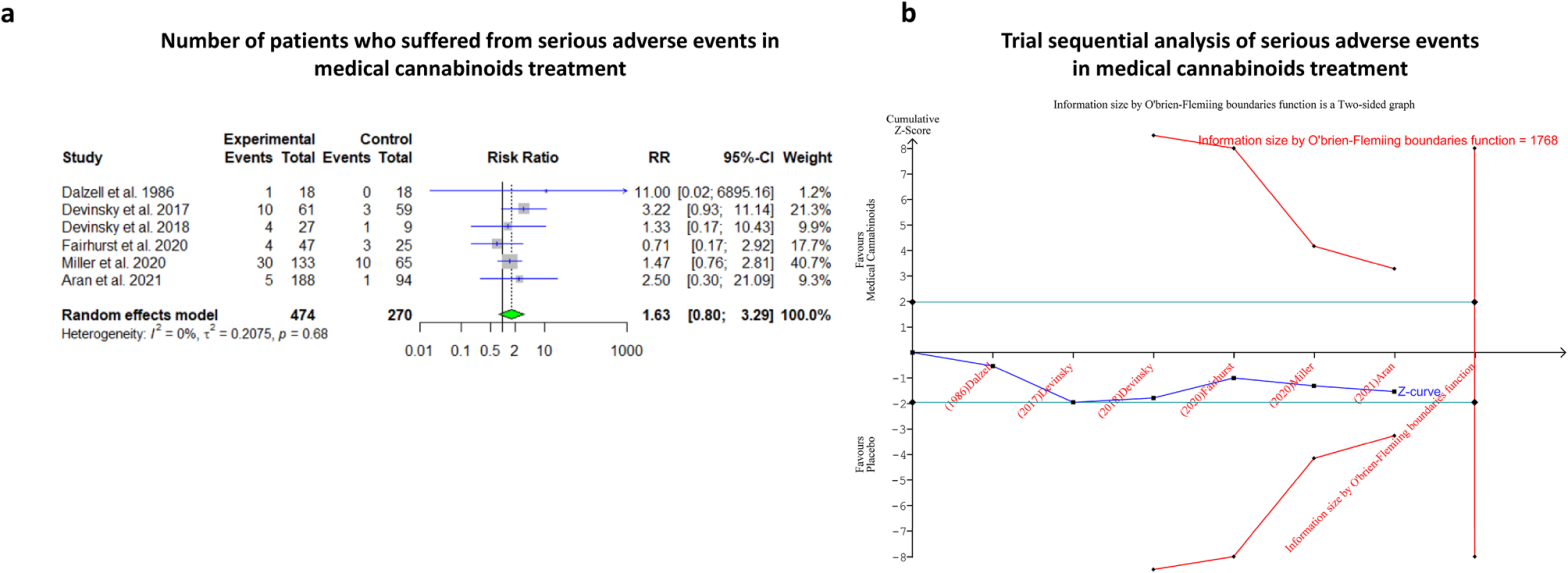
Serious adverse events in medical cannabis treatment. (**a**) Meta-analysis: serious adverse events in medical cannabinoids treatment (*p*-value = 0.14). (**b**) Trial seqaential analysis of serious adverse events in medical cannabinoids treatment, yielding information size of 1,768 participants for reaching statistical significance. Current evidence suggests *p*-value = 0.12 based on trial sequential analysis calculation.

Six studies, including 513 patients, reported on serious adverse events during the trials of treatment in MCs: three of them involved CBD, one study involved a CBD:THC mixture in ratio 20:1, respectively one study involved nabilone, the remaining study involved nabiximols. The pooled analysis suggests that MCs exposure may increase the risk for serious adverse events in comparison to control groups (RR = 1.59, 95% CI [0.85–2.98], *p*-value = 0.18, I^2^ = 0%). In TSA, the O’brein-fleming boundaries were not crossed, and the estimated IS to reach conclusive results in the a meta-analysis is 1768 randomized patients (Figs. 3A–B).

**Figure 4:**
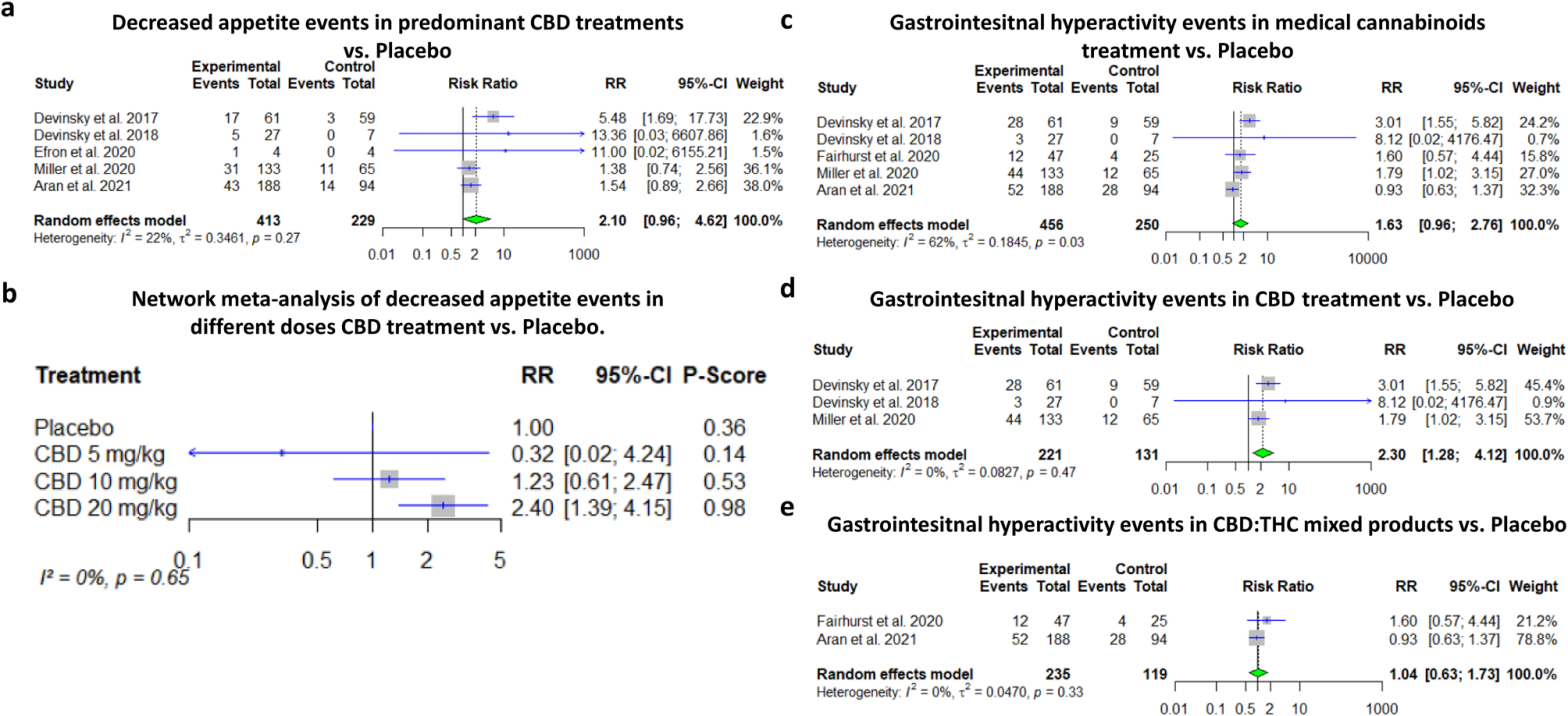
Decreased appetite in CBD treatments and other gastrointestinal events in medical cannabinoids treatment. (**a**) Meta-analysis: decreased appetite events in CBD treatment vs. Placebo (*p*-value = 0.06). (**b**) Network Meta-analysis: decreased appetite events in three doses of CBD treatment vs. Placebo (Aran et al. was excluded since only the mean dosage was reported and for most participants the goal dosage 10 mg/kg was not achieved). (**c**) Meta-analysis: Gastrointesitnal hyperactivity events in medical cannabinoids treatment vs. Placebo (*p*-value = 0.07). (**d**) Meta-analysis: Gastrointesitnal hyperactivity events in CBD treatment vs. Placebo (*p*-value = 0.005). (**e**) Meta-analysis: Gastrointesitnal hyperactivity events in CBD: THC mixed products vs. Placebo (*p*-value = 0.88).

The analysis concerning decreased appetite included all studies reported on these events, namely five studies of which four treated with pure CBD, while the remaining study treated with pre-dominant CBD product. The evidence suggests CBD may result in a relevant decrease in appetite (RR = 2.10, 95% CI [0.96–4.62], *p*-value = 0.07, I^2^ = 22%), although statistical significance was not reached. Since most of the data was focused on CBD products, subgroup analysis by composite was less feasible, and NMA was conducted. Based on the NMA, high dose of CBD (20 mg/kg/d) was associated with decreased appetite, while lower dose (10 mg/kg/d) might be also associated with decreased appetite although the effect size was quite limited and the results did not reach statistically significance (RR = 2.40, 95% CI [1.39–4.15], RR = 1.23 95% CI [0.61–2.47], respectively, I^2^ = 0%) (Fig. 4A–B). A similar tendency for other GI events (namely, diarrhea, upset stomach, nausea, retching and vomits) was found in the meta-analysis including three CBD studies, one study with an enriched CBD product and one study with nabiximols, although without reaching statistical significance (RR = 1.63, 95% CI [0.96–2.76], *p*-value = 0.07, I^2^ = 62%). Subgroup analysis revealed that CBD is associated with GI hyperactivity events (RR = 2.30, 95% CI [1.28–4.12], *p*-value = 0.005, I^2^ = 0%), while no evidence were found that mixed products are associated with these events (RR = 1.04, 95% CI [0.633–1.73], *p*-value = 0.88, I^2^ = 0%) (Fig. 4C–E).

Table 2 describes the association between medical cannabinoids and adverse mental events in the included studies. Due to varied incidence rates of these events, and multiple events reported on the same patient, no pooled analysis was performed. In almost all studies, the incidence rate for adverse mental events in the MC group was higher than the control group, regardless of indication and MC products composition. In four out eight studies the results are statistically significant.

**Table 2:** The association between medical cannabinoids and events of adverse mental events in the included studies.

Study	THC: CBD Ratio	Indication	Adverse mental events in MC group	N in MC group	Incidence rate in MC group	Adverse mental events control group	N in control group	Incidence rate in control group	Conditional maximum likelihood estimate of Rate Ratio	Confidence intervals*	*P*-value*
**Nabilone RCTs**											
Dalzell et al.^49^	NA	Chemotherapy-Induced Emesis	27	18	1.50	7	18	0.39	3.857143	1.639, 10.4	** < 0.0005**
Chan et al.^48^	NA	Chemotherapy-Induced Emesis	47	36	1.31	11	36	0.31	4.272727	2.184, 9.135	** > 0.0001**
**Mixed products**											
Fairhurst et al.^51^	2.7:2.5	Spasticity due to cerebral palsy or traumatic, non‐progressive CNS injury	6	47	0.13	2	25	0.08	1.595745	0.2853, 16.17	0.86
Aran et al.^53^	1:20	Autism spectrum disorder	591	188	3.14	257	94	2.73	1.149805	0.9915, 1.336	0.07
**CBD products**											
Devinsky et al.^28^	NA	Dravet syndrome	42	61	0.69	11	59	0.19	3.692996	1.869, 7.954	** > 0.0001**
Devinsky et al.^30^	NA	Dravet syndrome	14	27	0.52	4	7	0.57	0.907407	0.285, 3.786	1
Efron et al.^52^	NA	Severe behavioral problems with intellectual disability	4	4	1.00	1	4	0.25	4	0.3958, 197	0.375
Miller et al.^50^	NA	Dravet syndrome	72	133	0.54	20	65	0.31	1.759398	1.086, 2.952	**0.027**

*According to Fisher exact test.

Significant values in Bold.

Four studies, including 463 patients, reported outcomes of infections and pyrexia during the trials: three of them involved CBD, and one study involved nabiximols. The pooled analysis of infections demonstrated somewhat conflicting results, suggesting MCs treatment does not increase the risk for infections with very heterogeneous findings (RR = 0.94, 95%CI [0.47–1.76], *p*-value = 0.77, I^2^ = 75%). NMA revealed that neither a higher doses are not associated with change in risk for infections. On the other hand, a pooled analysis suggests that MCs may increase slightly the risk of pyrexia events (RR = 1.41, 95% CI [0.60–3.33], *p*-value = 0.44, I^2^ = 0%), although statistical significance was not reached (Supplementary Fig. 4a–c).

## Discussion

CBD results in an improvement in the epilepsy presentation of Dravet syndrome in all examined measurements. However, CBD probably results in decreased appetite and adverse mental events. Other MCs likely elevate the risk for adverse mental events as well. Previous systematic reviews based their findings on data retrieved from adults or included open-label and chart reviews in their analysis^31,32,55^. Till date, this is the first meta-analysis focusing on treatment with MCs in the pediatric population.

Most of the studies that reported outcomes related to seizure control in MCs treatment were assessed as being of high quality and included more than 450 pediatric patients. While CBD was associated with improvement in all examined measurements related to epilepsy control in Dravet syndrome, deepening the analysis reveals complexity in some of the results. Firstly, the analyses of reported seizure events under MC show that although CBD is associated with reducing seizure events, there is a suggesting trend that this improvement is annulled in products containing THC as well. This finding correlates with preclinical evidence, linking natural THC and synthetic cannabinoids with seizure induction via cannabinoid 1 receptor pathways^56^. Notably, the indications of the studies that used THC-containing products were different from epilepsy syndromes, and therefore the studied population was also different.

CBD products likely result in a 50% decreased risk of seizure events in children with epilepsy and improvement in CGIC. Although CBD likely improves better CGIC assessment than placebo, the range of reported assessments is wider in CBD groups because the disease was much worse under CBD treatment in some patients than baseline. Thus, these findings suggest that CBD treatment is expected to improve the clinical condition in most patients. However, in some cases, it may substantially exacerbate the epilepsy condition. The results suggest that CBD should be regarded as an adequate alternative treatment for uncontrolled epilepsy in Dravet syndrome and was approved in 2018 by FDA for this indication^57^. Nevertheless, the efficacy of CBD should be explicitly examined in the pediatric population in other presentations of epilepsy, such as Lennox-Gastaut syndrome, as previous studies did not publish specific results concerning this population. These findings align with previous meta-analyses for some similar outcomes which did not focus on the pediatric population^31,32^.

Two studies with a total of 48 patients have examined nabilone’s efficacy in CINV in children. The pooled analysis suggested nabilone results in fewer events of vomiting and retching compared to conventional antiemetic treatment. However, both studies were conducted in the 80’s and were judged to be at high risk of bias. Furthermore, the therapy options have been expanded during the last 30 years^58^. Therefore, despite the promising results, additional high-quality studies should compare nabilone efficacy with modern standard treatments. A similar interpretation was reflected in the adult population as well^59^.

Although the efficacy of MCs was associated with a clinical improvement in this meta-analysis, it showed that medical cannabinoids are associated with safety aspects, including SAE incidence, decrease in appetite and adverse mental events in the pediatric population. The pooled analysis on patients who suffered from SAEs was mainly focused on CBD due to limited data about other compounds. Notwithstanding, the current evidence does not suggest a difference between the different products as no heterogeneity was observed. The pooled analysis yielded a non-significant elevation of 1.63-fold risk, suggesting MCs exposure may increase the risk for serious adverse events compared to the reference groups. The absence of statistical significance may be derived from insufficient power, as indicated by trial sequential analysis calculation. These findings align with a recently published meta-analysis focusing on CBD and encompassing 710 participants from all ages reported^60^. Although further research is needed to establish this safety signal concerning children since pediatric pharmacotherapy should be conducted with extra precaution, clinicians should be aware of this disturbing finding.

The analysis further showed that CBD treatment is not statistically associated with decreased appetite events with slight heterogeneity between studies. NMA suggested that different dosages might explain heterogeneity, and demonstrated that 20 mg/kg/d CBD results in a significant, elevated risk for decreased appetite, whereas 10 mg/kg/d might result in a mild elevation in the risk, yet not reached statistical significance. CBD’s pharmacological and clinical effects are not well characterized, especially in the pediatric population. Previous studies reported that CBD has opposite impacts to those triggered by THC, as it has anti-anxiolytic, anti-epileptic, and antipsychotic properties^60^. While there is conflicting evidence regarding the effects of CBD on food intake and appetite, the meta-analysis suggests that CBD has decreased appetite, although no CBD biological mechanism was fully deciphered^61–63^.

This finding is significant in children’s therapy, as decreased appetite may compromise children’s development. Furthermore, epilepsy syndromes and autism are associated with growth impairment and feeding problems^64,65^. Thus healthcare providers should be familiar with this risk and follow the patient’s nutrient adequacy and physical development during CBD treatment. CBD treatment may also result in additional GI adverse events, such as GI hyperactivity, strengthening the concept of nutritional vigilance in CBD treatment.

The pooled analysis presented in Table 2 suggests that MCs likely result in an increased risk for adverse mental events. This increase was observed in all included studies, regardless of the cannabinoids composition or indication. However, it seems higher in nabilone studies than in studies utilizing mixed THC-CBD or CBD products. THC is well known for its psychoactive and negative cognitive effects, and therefore these findings are expected in THC derivatives. Furthermore, this pooled data show that adverse mental events in children also appear under exposure to CBD, known for its anxiolytic effect. Although some of CBD’s effects are not well deciphered, evidence shows that it may change endocannabinoid system tone by inhibiting endocannabinoids metabolism an allosteric modulator and an indirect antagonist of the cannabinoid receptors, in the endocannabinoid system^66,67^. It may also function as agonist of nonselective cation channels and transient potential vanilloid receptor types 1 and 2. CBD administration was also shown to be associated with increased levels of biomarkers of neuronal differentiation (DCX + cells) and hippocampal neurogenesis (BrdU + NeuN + cells) in several models in mice in low doses of CBD^68^.

Nevertheless, children and adolescents may be affected by cannabinoids other than adults, as cannabinoids exposure may pose long-term consequences in this population^68–70^. Previous reports in animals shows that endocannabinoids levels in the various locations in the CNS are significantly higher during juvenile and pubescence, and drastically decreases in adulthood. The cannabinoid receptors are also notably abundant in the corticolimbic brain regions in adolescent rats. Further studies showed that cannabinoids exposure during developmental stages impairs learning and memory development and was associated with anxiety. The permanent consequences of cannabinoids exposure are still unclear, as timing and duration of exposure might play a crucial role in its long-term trajectory. Nevertheless, this evidence suggests that modulation of the endocannabinoid system in children and adolescents alters functional and structural plasticity in the CNS. Till date, no controlled studies were conducted in children for long periods, and therefore this meta-analysis could not address the long-term effects of cannabinoids on development. One can assume the exogeneous addition of cannabinoids, such as described in this meta-analysis may influence brain development and behavior, and may pose long-term effects, especially when mental signs and symptoms persist^68,69^.

The potential harmful effects of MC suggest that healthcare providers should be aware of possible adverse mental events regardless of its formula and indications for treatment.

CBD specifically was previously associated with infections in RCTs. However, this meta-analysis suggests that CBD or other MCs do not increase the number of diseases events. On the other hand, the analysis showed that pyrexia events, usually related to infections, were more likely under CBD, although the results were not significant. Since these results are inconclusive, additional research is needed to determine whether CBD is associated with infections and pyrexia.

This meta-analysis has addressed the prominent safety and efficacy outcomes in MCs treatment. However, it still held several weaknesses: the data are still somewhat limited, as the studied population includes less than 650 patients. Another limitation of this study is the observational nature of the data analysis. The data about medical cannabinoids treatment in children is limited, originated in relatively small number of small studies divided to several medical indications. Since every study was expected to measure outcomes differently, prior selection of specific questions for investigation would have led to missing important trends and implications. Although significant findings are shown in this analysis, further research and analysis should be done in the future for strengthening the observations demonstrated in this work.

In this meta-analysis, the varied follow up periods might contribute to the heterogeneity, ranging from 3 to 14 weeks in the efficacy analysis and 8–18 weeks in the safety analysis, and two studies period were done within “two cycles of chemotherapy”. Since the number of included studies is relatively small and mostly concentrated in the range of 9–18 weeks, it is hard to determine whether the follow up period was a contributing factor to the heterogeneity. Therefore, this meta-analysis used random effects model and Sidik-Jonkman conservative method for taking into consideration the designed heterogeneity, which came into effect only in some of the examined outcomes^71^. Additionally, data from longer follow up periods is still needed for evaluation the long term effects of medical cannabinoids on the pediatric population.

Furthermore, potential important indications, such as IBD, were not retained due to a lack of relevant and compatible data. The statistical power might be inadequate for some outcomes, and not enough information is available to determine the relevance of these analyses. Although essential information has already been reported, further high-quality studies are needed to clarify the remaining uncertainties regarding efficacy and safety aspects in MCs treatment in children. In addition, the medical assessment of some Dravet cases, the most investigated indication in this meta-analysis, might be difficult to judge due to severely impaired functionality. Nevertheless, CBD demonstrated efficacy as an anticonvulsant in Dravet syndrome treatment in the pediatric population, while its effects on appetite and physical development should be considered. In addition, MCs increase adverse mental events and should be taken into consideration before and during treatment.

## Conclusions

CBD demonstrated efficacy in epilepsy treatment in the pediatric population. Nevertheless, exposure to MCs might be associated with SAEs. Healthcare providers should follow appetite and physical development in children exposed to CBD. In addition, MCs (CBD and THC analogues) are probably associated with adverse mental events. These effects should be taken into consideration before and during treatment, and should be examined in studies conducted for the long term.

## Supplementary Information

Supplementary Information.

## Abbreviations

ASD: Autism spectrum disorder
CBD: Cannabidiol
CGIC: Caregiver global impression of change
CINV: Chemotherapy-induced nausea and vomiting
CI: Confidence of intervals
IBD: Inflammatory bowel disease
IS: Information size
MCs: Medical cannabinoids
NMA: Network meta-analysis
RCT: Randomized control trial
RR: Relative risk
SAE: Serious adverse events
THC: D-9-Tetrahydrocannabinol
