# Supplementary material for: Efficacy and safety of medical cannabinoids in children: a systematic review and meta-analysis
Source: Sci Rep. 2021 Dec 6;11:23462. doi: 10.1038/s41598-021-02770-6 (PMC8648720; doi:10.1038/s41598-021-02770-6)
Supplement: Supplementary file 1 — Supplementary Information. [file 41598_2021_2770_MOESM1_ESM.docx]

**Supplementary Online Content**

**Supplementary Table S1: PRISMA-IPD Checklist of Items to Include When Reporting a Systematic Review and Meta-analysis of Individual Participant Data**

**Supplementary Method: Meta-analysis protocol**

**Supplementary Method: Strategy search**

**Supplementary Method: variables for which data were extracted in extraction form**

**Supplementary Method: Terms and aggregated events**

**Supplementary Method: Scripts in R**

**Supplementary Table S2: Summary findings table**

**Supplementary Figure S1: Search algorithm diagram**

**Supplementary Figure S2: Quality assessment of the included studies according to RoB-2 scale**

**Supplementary Figure S3: Efficacy of nabilone versus conventional control as an antiemetic treatment**

**Supplementary Figure S4a-c: Events of infections and pyrexia under medical cannabinoids treatment**

**Supplementary Table S1: PRISMA-IPD Checklist of Items to Include When Reporting a Systematic Review and Meta-analysis of Individual Participant Data**

| **Section/topic** | **#** | **Checklist item** | **Reported on page #** |
| --- | --- | --- | --- |
| **TITLE** | | |  |
| Title | 1 | Identify the report as a systematic review, meta-analysis, or both. | 1 |
| **ABSTRACT** | | |  |
| Structured summary | 2 | Provide a structured summary including, as applicable: background; objectives; data sources; study eligibility criteria, participants, and interventions; study appraisal and synthesis methods; results; limitations; conclusions and implications of key findings; systematic review registration number. | 3 |
| **INTRODUCTION** | | |  |
| Rationale | 3 | Describe the rationale for the review in the context of what is already known. | 4-5 |
| Objectives | 4 | Provide an explicit statement of questions being addressed with reference to participants, interventions, comparisons, outcomes, and study design (PICOS). | 5 |
| **METHODS** | | |  |
| Protocol and registration | 5 | Indicate if a review protocol exists, if and where it can be accessed (e.g., Web address), and, if available, provide registration information including registration number. | 5-6 |
| Eligibility criteria | 6 | Specify study characteristics (e.g., PICOS, length of follow-up) and report characteristics (e.g., years considered, language, publication status) used as criteria for eligibility, giving rationale. | 6 |
| Information sources | 7 | Describe all information sources (e.g., databases with dates of coverage, contact with study authors to identify additional studies) in the search and date last searched. | 6 |
| Search | 8 | Present full electronic search strategy for at least one database, including any limits used, such that it could be repeated. | 6+  Supplementary Method |
| Study selection | 9 | State the process for selecting studies (i.e., screening, eligibility, included in systematic review, and, if applicable, included in the meta-analysis). | 6-7 |
| Data collection process | 10 | Describe method of data extraction from reports (e.g., piloted forms, independently, in duplicate) and any processes for obtaining and confirming data from investigators. | 6-7 |
| Data items | 11 | List and define all variables for which data were sought (e.g., PICOS, funding sources) and any assumptions and simplifications made. | 7 |
| Risk of bias in individual studies | 12 | Describe methods used for assessing risk of bias of individual studies (including specification of whether this was done at the study or outcome level), and how this information is to be used in any data synthesis. | 6-7 |
| Summary measures | 13 | State the principal summary measures (e.g., risk ratio, difference in means). | 7+8 |
| Synthesis of results | 14 | Describe the methods of handling data and combining results of studies, if done, including measures of consistency (e.g., I^2^) for each meta-analysis. | 8 |

| **Section/topic** | **#** | **Checklist item** | **Reported on page #** |
| --- | --- | --- | --- |
| Risk of bias across studies | 15 | Specify any assessment of risk of bias that may affect the cumulative evidence (e.g., publication bias, selective reporting within studies). | 8 |
| Additional analyses | 16 | Describe methods of additional analyses (e.g., sensitivity or subgroup analyses, meta-regression), if done, indicating which were pre-specified. | 8+9 |
| **RESULTS** | | |  |
| Study selection | 17 | Give numbers of studies screened, assessed for eligibility, and included in the review, with reasons for exclusions at each stage, ideally with a flow diagram. | Supplementary Figure 1 |
| Study characteristics | 18 | For each study, present characteristics for which data were extracted (e.g., study size, PICOS, follow-up period) and provide the citations. | Table 1 |
| Risk of bias within studies | 19 | Present data on risk of bias of each study and, if available, any outcome level assessment (see item 12). | Supplementary Figure 2 |
| Results of individual studies | 20 | For all outcomes considered (benefits or harms), present, for each study: (a) simple summary data for each intervention group (b) effect estimates and confidence intervals, ideally with a forest plot. | Figures 1-3, Table 2,  Supplementary Figure 3  Supplementary Figure 4 |
| Synthesis of results | 21 | Present results of each meta-analysis done, including confidence intervals and measures of consistency. | 8-9  Figures 1-3  Supplementary Figure 3  Supplementary Figure 4 |
| Risk of bias across studies | 22 | Present results of any assessment of risk of bias across studies (see Item 15). | Not applicable – less than 10 studies |
| Additional analysis | 23 | Give results of additional analyses, if done (e.g., sensitivity or subgroup analyses, meta-regression [see Item 16]). | 10-11  Figures 1-3  Table 2  Supplementary Figure 3  Supplementary Figure 4 |
| **DISCUSSION** | | |  |
| Summary of evidence | 24 | Summarize the main findings including the strength of evidence for each main outcome; consider their relevance to key groups (e.g., healthcare providers, users, and policy makers). | 12-16 |
| Limitations | 25 | Discuss limitations at study and outcome level (e.g., risk of bias), and at review-level (e.g., incomplete retrieval of identified research, reporting bias). | 15 |
| Conclusions | 26 | Provide a general interpretation of the results in the context of other evidence, and implications for future research. | 16 |
| **FUNDING** | | |  |
| Funding | 27 | Describe sources of funding for the systematic review and other support (e.g., supply of data); role of funders for the systematic review. | 1 |

**Supplementary Method: Meta-analysis protocol**

| Assessing the safety and efficacy of medical cannabis in treatment of children: A systematic review and meta-analysis | Title of the review |
| --- | --- |
| Nir Treves | First reviewer (s) |
| Noa Mor, Nir Treves and Ilan Matok, PhD | Team of reviewers |
| Ilan Matok, PhD | Supervisor/Project PI |
| Nir Treves, PhD candidate, Noa Mor, Pharm D candidate, Ilan Matok, Msc Pharm, PhD | Clinical Portfolio Group |

| Support- please state if advice/training or personnel required at each stage | |
| --- | --- |
| Advice and Training with Tomer Ben Shushan, information scientist and librarian in Medical Library of the Hebrew University in Jerusalem.  Ilan Matok, PhD, who has a vast experience with supervision and leading systematic reviews and meta-analyses. | SR overview |
| Noa mor and Nir Treves under the supervision of Dr. Ilan Matok. | Protocol development |
| Noa Mor and Nir Treves  The literature searching is conducted with the assistance of Tomer Ben Shoshan. | Literature searching |
| Noa Mor and Nir Treves.  Nir Treves has previously performed a systematic review and a meta-analysis and conducted quality appraisal.  Advice gained from Dr. Ilan Matok and from reading materials in the field. | Quality appraisal |
| Noa Mor and Nir Treves.  Within previous meta-analysis Nir Treves has extracted data from the literature.  Advice gained from Dr. Ilan Matok and from the literature in this field. | Data Extraction |
| Noa Mor and Nir Treves.  Nir Treves has previously performed a systematic review and a meta-analysis.  Advice gained from Dr. Ilan Matok and from the literature in the field. | Synthesis |
| Noa Mor and Nir Treves.  Nir Treves has previously performed a systematic review and a meta-analysis.  Noa Mor has some experience in literature reviews.  Advice gained from Dr. Ilan Matok and from the literature in the field. | Writing up |

| 1. **Background to review** Brief introduction to the subject of the review, including rationale for undertaking the review and overall aim | To date, Cannabinoids are still considered a controlled substance, which is used for medical purposes. As their beneficial effects are gradually revealed and its therapeutic indications are developing, data on their safety is still limited. In children the data on their safety and efficacy are even scarcer. We will review the aggregated safety data which has been collected till day to reveal the main safety issues concerning the medical cannabis treatment in children. |
| --- | --- |
| **2. Specific objectives** |  |
| **3. a) Criteria for including studies in the review** If the PICOS format does not fit the research question of interest, please split up the question into separate concepts and put one under each heading |  |
| **i. Population, or participants**  **and conditions of**  **interest** | Children under the age of 18 that were treated with cannabinoids for any indication. No language limitation will be implemented. |
| **Interventions or**  **exposures** | Children under the treatment of medical cannabis and its derivatives. |
| **Comparisons or control**  **groups** | Placebo or conventional treatment. |
| **Outcomes of interest** | Safety issues, i.e adverse events as well as efficacy indices. |
| **Settings** | No limitations. |
| **Study designs** | Any study design: we expect to find more RCT's rather than observational studies. |
| **3. b) Criteria for excluding studies not covered in inclusion criteria** Any specific populations excluded, date range, language, whether abstracts or full text available, etc | Adults, (Age>18)  Availability of abstracts only.  Case reports or case series.  Studies without comparison group. |
| **4. Search methods:**   - **Electronic databases** Please list all databases that are to be searched and include the interface (eg NHS, EBSCO, etc) and date ranges searched for each - **Other methods used for identifying relevant research** ie contacting experts and reference checking - **Journals hand searched** If any are to be hand searched, please list which journals and date searched from, including a rationale. | Pubmed, Embase, Clinicaltrials, Cochrane.  No date limitations.  In eligible articles we will review its reference in order to find more relevant studies. |

| **5. Methods of review** | |
| --- | --- |
| **Details of methods** Number of reviewers, how agreements to be reached and disagreements dealt with, etc. | Two main reviewers and a third to resolve any disagreements  Main reviewers: Nir Treves and Noa Mor.  Third reviewer PhD. Ilan Matok  Agreed data to be extracted and terminology be clarified beforehand. |
| **Quality assessment** Tools or checklists used with references or URLs | Cochrane risk-of-bias tool for randomized trials  Newcastle Ottawa for observational studies/  Robins for observational studies |
| **Data extraction** What information is to be collected on each included study. If databases or forms on Word or Excel are used and how this is recorded and by how many reviewers | Study type, indications examined under the study, study population, year of publish, number of participants, range of ages, Safety information such as figures of adverse events efficacy indices adjustments for confounders, funding, Substances under examinations, route of administration, follow up period.  Data extraction with Rayyan internet site.  Two reviewers will perform the extractions.  Reviews' reference will be screened for additional eligible studies. |
| **Narrative synthesis** Details of what and how synthesis will be done | Joint synthesis by two reviewers.  CMA or R will be used for synthesis, based on the complexity of the results. |
| **Meta-analysis** Details of what and how analysis and testing will be done. If no meta-analysis is to be conducted, please give reason | Although a meta-analysis is planned, the extent of the meta-analysis will be determined after data is extracted and made available from the systematic review.  Heterogeneity issues will be explored and dealt with based on the results (Random effect model, as well as subgroup analysis, meta regression and qualitative analysis of heterogeneity). R scripts for meta-analysis will be utilized.  Based on the results we will consider meta regression |

| 6. Presentation of results | |
| --- | --- |
| **Additional material** Summary tables, flowcharts, etc, to be included in the final paper | Table 1 for articles  Algorithm for search  Protocol  Funnel plot  Forest plots Figures showing the pooled results (main analysis as well as sub-analysis) |
| **Outputs from review** Papers and target journals, conference presentations, reports, etc | Unknown at this stage |

| **7. Timeline for review – when do you aim to complete each stage of the review** | |
| --- | --- |
| **Protocol** | **1 months** |
| **Literature searching** | **2 months** |
| **Quality appraisal** | **2 months** |
| **Data extraction** | **2 months** |
| **Synthesis** | **2 months** |
| **Writing up** | **2 months** |

**Supplementary Method: Strategy search**

Embase syntax:

('medical marijuana':ti,ab,kw OR 'cannabinoid':ti,ab,kw OR '1 butyl 3 (1 naphthoyl)indole':ti,ab,kw OR '1 deoxy 3 (1,1 dimethylbutyl) delta8 tetrahydrocannabinol':ti,ab,kw OR '11 hydroxydronabinol':ti,ab,kw OR '2 arachidonoylglycerol':ti,ab,kw OR '2 methyl 3 (1 naphthoyl) 1 propylindole':ti,ab,kw OR '2,3 dihydro 5 methyl 3 (morpholinomethyl) 6 (1 naphthoyl)pyrrolo[1,2,3 de][1,4]benzoxazine':ti,ab,kw OR '20 cyano n (2 hydroxy 1 methylethyl) 16,16 dimethylarachidonamide':ti,ab,kw OR '3 (1 naphthoyl) 1 pentylindole':ti,ab,kw OR '3 (2 iodo 5 nitrobenzoyl) 1 (1 methyl 2 piperidinylmethyl)indole':ti,ab,kw OR '3 (5 cyano 1,1 dimethylpentyl) 1 (4 morpholinobutyryloxy) delta8 tetrahydrocannabinol':ti,ab,kw OR '3 hydroxy delta9 tetrahydrocannabinol':ti,ab,kw OR '4 (1,1 dimethylheptyl) 1,2,3,4,5,6 hexahydro 2,3 dihydroxy 6 (3 hydroxypropyl)biphenyl':ti,ab,kw OR '4 [4 (1,1 dimethylheptyl) 2,6 dimethoxyphenyl] 6,6 dimethylbicyclo[3.1.1]hept 2 en 2 ylmethanol':ti,ab,kw OR '5 (1,1 dimethylheptyl) 2 (3 hydroxy 7 hydroxymethyldecahydro 1 naphthyl)phenol':ti,ab,kw OR '7 (6a,7,10,10a tetrahydro 1 hydroxy 6,6,9 trimethyl 6h benzo[c]chromen 3 yl) 5 heptynenitrile':ti,ab,kw OR 'anandamide':ti,ab,kw OR 'cannabichromene':ti,ab,kw OR 'cannabidiol':ti,ab,kw OR 'cannabidiol derivative':ti,ab,kw OR 'cannabidiol dimethyl ether':ti,ab,kw OR 'cannabidiol methyl ether':ti,ab,kw OR 'cannabidivarin':ti,ab,kw OR 'cannabielsoin':ti,ab,kw OR 'cannabigerol':ti,ab,kw OR 'cannabinoid derivative':ti,ab,kw OR 'cannabinol':ti,ab,kw OR 'cannabinol derivative':ti,ab,kw OR 'cannabis':ti,ab,kw OR 'cannabis derivative':ti,ab,kw OR 'deacetyllevonantradol':ti,ab,kw OR 'delta8 tetrahydrocannabinol':ti,ab,kw OR 'delta8 tetrahydrocannabinol derivative':ti,ab,kw OR 'delta9(11) tetrahydrocannabinol':ti,ab,kw OR 'dexanabinol':ti,ab,kw OR 'dextronantradol':ti,ab,kw OR 'dronabinol':ti,ab,kw OR 'ehp101':ti,ab,kw OR 'endocannabinoid':ti,ab,kw OR 'lenabasum':ti,ab,kw OR 'levonantradol':ti,ab,kw OR 'medical cannabis':ti,ab,kw OR 'methanandamide':ti,ab,kw OR 'n [6 (6a,7,10,10a tetrahydro 6,6,9 trimethyl 6h benzo[c]chromen 3 yl) 4 hexenyl]ethanesulfonamide':ti,ab,kw OR 'n oleoylethanolamine':ti,ab,kw OR 'nabiximols':ti,ab,kw OR 'nantradol':ti,ab,kw OR 'noladin ether':ti,ab,kw OR 'palmidrol':ti,ab,kw OR 'tetrahydrocannabinol':ti,ab,kw

Pubmed syntax:

Search ((((((medical marijuana• OR cannabinoid OR .1 butyl 3 (1 naphthoyl)indole• OR .1 deoxy 3 (1,1 dimethylbutyl) delta8 tetrahydrocannabinol• OR .11 hydroxydronabinol• OR .2 arachidonoylglycerol• OR .2 methyl 3 (1 naphthoyl) 1 propylindole• OR .2,3 dihydro 5 methyl 3 (morpholinomethyl) 6 (1 naphthoyl)pyrrolo[1,2,3 de][1,4]benzoxazine• OR .20 cyano n (2 hydroxy 1 methylethyl) 16,16 dimethylarachidonamide• OR .3 (1 naphthoyl) 1 pentylindole• OR .3 (2 iodo 5 nitrobenzoyl) 1 (1 methyl 2 piperidinylmethyl)indole• OR .3 (5 cyano 1,1 dimethylpentyl) 1 (4 morpholinobutyryloxy) delta8 tetrahydrocannabinol• OR .3 hydroxy delta9 tetrahydrocannabinol• OR .4 (1,1 dimethylheptyl) 1,2,3,4,5,6 hexahydro 2,3 dihydroxy 6 (3 hydroxypropyl)biphenyl• OR .4 [4 (1,1 dimethylheptyl) 2,6 dimethoxyphenyl] 6,6 dimethylbicyclo[3.1.1]hept 2 en 2 ylmethanol• OR .5 (1,1 dimethylheptyl) 2 (3 hydroxy 7 hydroxymethyldecahydro 1 naphthyl)phenol• OR .7 (6a,7,10,10a tetrahydro 1 hydroxy 6,6,9 trimethyl 6h benzo[c]chromen 3 yl) 5 heptynenitrile• OR anandamide OR cannabichromene OR cannabidiol OR oecannabidiol derivative• OR oecannabidiol dimethyl ether• OR oecannabidiol methyl ether• OR cannabidivarin OR cannabielsoin OR cannabigerol OR oecannabinoid derivative• OR cannabinol OR oecannabinol derivative• OR cannabis OR oecannabis derivative• OR deacetyllevonantradol OR oedelta8 tetrahydrocannabinol• OR oedelta8 tetrahydrocannabinol derivative• OR oedelta9(11) tetrahydrocannabinol• OR dexanabinol OR dextronantradol OR dronabinol OR ehp101 OR endocannabinoid OR lenabasum OR levonantradol OR oemedical cannabis• OR methanandamide OR oen [6 (6a,7,10,10a tetrahydro 6,6,9 trimethyl 6h benzo[c]chromen 3 yl) 4 hexenyl]ethanesulfonamide• OR oen oleoylethanolamine• OR nabiximols OR nantradol OR oenoladin ether• OR palmidrol OR tetrahydrocannabinol OR oetetrahydrocannabinol derivative• OR oetetrahydrocannabinolic acid• OR virodhamine))) OR ((("Cannabinoids"[Mesh]) OR "Medical Marijuana"[Mesh]) OR "Cannabis"[Mesh]))) AND ((((adolescence[Title/Abstract] OR adolescent[Title/Abstract] OR childhood[Title/Abstract] OR newborn[Title/Abstract] OR pediatric[Title/Abstract] OR paediatric[Title/Abstract] OR child[Title/Abstract] OR infant[Title/Abstract] OR toddler[Title/Abstract] OR teenage[Title/Abstract] OR young[Title/Abstract] OR baby[Title/Abstract] OR juvenile[Title/Abstract]))) OR ((("Adolescent"[Mesh]) OR "Child"[Mesh]) OR "Infant"[Mesh]))) AND ((((efficacy[Title/Abstract] OR safety[Title/Abstract] OR oetreatment outcome•[Title/Abstract] OR oeside effect•[Title/Abstract] OR oeadverse reaction•[Title/Abstract] OR oeadverse drug reaction•[Title/Abstract] OR risk[Title/Abstract] OR oetreatment response•[Title/Abstract] OR oeclinical effectiveness•[Title/Abstract] OR oeadverse event•[Title/Abstract]))) OR ((("Treatment Outcome"[Mesh]) OR "Patient Safety"[Mesh]) OR "Drug-Related Side Effects and Adverse Reactions"[Mesh]))

Cochrane syntax:

Search Name: "medical marijuana" OR cannabis OR cannabinoids AND adolescence OR adolescent OR childhood OR newborn OR pediatric OR paediatric OR child OR infant OR toddler OR teenage OR young OR baby OR juvenile in Title Abstract Keyword AND efficacy OR safety OR “treatment outcome” OR “side effect” OR “adverse reaction” OR “adverse drug reaction” OR risk OR “treatment response” OR “clinical effectiveness” OR “adverse event” in Title Abstract Keyword (Word variations have been searched) ID Search

#1 "medical marijuana" OR cannabis OR cannabinoids AND adolescence OR adolescent OR childhood OR newborn OR pediatric OR paediatric OR child OR infant OR toddler OR teenage OR young OR baby OR juvenile:ti,ab,kw AND efficacy OR safety OR “treatment outcome” OR “side effect” OR “adverse reaction” OR “adverse drug reaction” OR risk OR “treatment response” OR “clinical effectiveness” OR “adverse event”:ti,ab,kw (Word variations have been searched)

**Supplementary** **Method: variables for which data were extracted in extraction form**

**General information:** Paper title, authors, year of publishment.

**Study focus and methods:** Aim, Context, Research Questions, Method, Sample size**,** characters of Participants (age, diagnosis, adjusted variables if applicable, additional pharmacotherapy).

**Interventions:** Number of exposed participants, exposure material, route of administration, Dose of cannabinoid

**Comparators:** Number of exposed participants, control material, route of administration, dose of control.

**Study design:** design, follow up period, outcomes measured in the study, statistical analysis**,** Approaches to data analysis and interpretation, endpoints, withdrawal rate,

**Results: results of efficacy and safety outcomes,** method of measurements, specification level of safety information.

**Miscellaneous**: quality score and funding.

**Supplementary Method: Terms and aggregated events**

1. Serious adverse event (SAE) - SAE according to clinical trials glossary is an adverse event that results in death, is life-threatening, requires inpatient hospitalization or extends a current hospital stay, results in an ongoing or significant incapacity or interferes substantially with normal life functions, or causes a congenital anomaly or birth defect. Medical events that do not result in death, are not life-threatening, or do not require hospitalization may be considered serious adverse events if they put the participant in danger or require medical or surgical intervention to prevent one of the results listed above.
2. The outcome Adverse mental events include the following events: fatigue, somnolence, unresponsive to stimuli, lethargy, sedation, drowsiness, dizziness, vagueness, lightheadedness, and flat affect, Suicidal thoughts, Concentration problems, Memory problems, weakness, unsteadiness, disorientation, mood swings, motivational problems, anxiety, depression, tiredness, confusion, nervousness, restlessness, excitability, irritability, hysteria crying, aggression, abnormal behavior, psychomotor hyperactivity, hallucinations, elevation of mood, mood changes, euphoria.
3. Reported events in the outcome of infections included the following: pneumonia, viral infection coxsackie viral infection, gastroenteritis, sepsis, adenovirus infection, laryngitis, respiratory tract infection, viral upper respiratory tract infection, nasopharyngitis, upper respiratory tract infection, urinary tract infection, upper respiratory tract, viral gastroenteritis, viral infection, streptococcal pharyngitis, adenoviral pneumonia, sinusitis.

**Supplementary Method: Scripts in R**

#meta analysis serious adverse events:

Serious.bin <- metabin(Ee,

Ne, Ec, Nc, data = EXCEL_FOR_R_serious_cases_2021, studlab = paste(Author), comb.fixed = FALSE,

comb.random = TRUE, method.tau = "SJ", hakn = FALSE, incr = 0.1, sm = "RR")

summary(Serious.bin)

#forest serious adverse events:

forest(Serious.bin, col.inside = "blue", col.study = "blue", col.diamond = "green", xlim = c(0.01, 1000))

#meta analysis decreased appetite:

decreased_appetite_no_0.bin <- metabin(Ee, Ne, Ec,Nc, data = EXCEL_FOR_R_decreased_appetite_no_thc, studlab = paste(Author), comb.fixed = FALSE,comb.random = TRUE, method.tau = "SJ", hakn = FALSE, incr = 0.1, sm = "RR")

summary(decreased_appetite_no_0.bin)

#forest decrease appetite

forest(decreased_appetite_no_0.bin, col.inside = "blue", col.study = "blue", col.diamond = "green", xlim = c(0.01, 1000))

#NMA decrease appetite

decreased_appetite.NMA <- pairwise(treat = list(Treatment1,Treatment2, Treatment3, Treatment4), event = list(Case1, Case2, Case3, Case4), n = list(N1, N2, N3, N4), data = NMA_decreased_appetite2, sm = "RR")

NMA_decrease <- netmeta(TE, seTE, treat1, treat2, studlab, data = decreased_appetite.NMA, comb.fixed = FALSE, comb.random = TRUE)

#Pscore

NMArank <- netrank(NMA_decrease, small.values = "bad")

#forest decrease appetite - NMA

forest(NMA_decrease, reference.group="Placebo", sortvar = c(3,4,2,1), drop.reference.group=FALSE, rightcols=c("effect", "ci", "Pscore"), rightlabs="P-Score", small.values="bad",col.inside = "blue", col.study = "blue", col.diamond = "green", xlim = c(0.1, 5), smlab = "Placebo vs. CBD doses of 10mg/kg and 20mg/kg\n\n", text.addline1 = paste("\n\n\n I² = 0%, p = 0.65"), ff.addline = 3)

# GI hyperactivity:

GI_tract.bin <- metabin(Ee, Ne, Ec, Nc, data = EXCEL_FOR_R_hyperactivity_GI_updated, studlab = paste(author), comb.fixed = FALSE, comb.random = TRUE, method.tau = "SJ", hakn = FALSE, incr = 0.1, sm = "RR")

summary(GI_tract.bin)

#forest GI tract

forest(GI_tract.bin, col.inside = "blue", col.study = "blue", col.diamond = "green", xlim = c(0.01, 10000))

#forest GI tract- CBD:THC mixed products

GI_tract_THC.bin <- metabin(Ee, Ne, Ec, Nc, data = EXCEL_FOR_R_hyperactivity_GI_THC, studlab = paste(author), comb.fixed = FALSE, comb.random = TRUE, method.tau = "SJ", hakn = FALSE, incr = 0.1, sm = "RR")

summary(GI_tract_THC.bin)

forest(GI_tract_THC.bin, col.inside = "blue", col.study = "blue", col.diamond = "green", xlim = c(0.01, 1000))

# forest GI tract- CBD- only

GI_tract_CBD.bin <- metabin(Ee, Ne, Ec, Nc, data = EXCEL_FOR_R_hyperactivity_GI_CBD, studlab = paste(author), comb.fixed = FALSE, comb.random = TRUE, method.tau = "SJ", hakn = FALSE, incr = 0.1, sm = "RR")

summary(GI_tract_CBD.bin)

#pyrexia:

pyrexia.bin <-metabin(Ee, Ne, Ec,Nc, data = EXCEL_FOR_R_pyrexia, studlab = paste(author), comb.fixed = FALSE,comb.random = TRUE, method.tau = "SJ", hakn = FALSE, incr = 0.1, sm = "RR")

summary(pyrexia.bin)

#forest pyrexia

forest(pyrexia.bin, col.inside = "blue", col.study = "blue", col.diamond = "green", xlim = c(0.01, 50))

#infections:

infect.bin<-metabin(Ee, Ne, Ec,Nc, data = EXCEL_FOR_R_infections, studlab = paste(author), comb.fixed = FALSE,comb.random = TRUE, method.tau = "SJ", hakn = FALSE, incr = 0.1, sm = "RR")

summary(infect.bin)

#forest infections:

forest(infect.bin, col.inside = "blue", col.study = "blue", col.diamond = "green", xlim = c(0.01, 100))

#Infections NMA:

NMA_infections <- pairwise(treat = list(Treatment2, Treatment3, Treatment4), event = list(Case2, Case3, Case4), n = list(N2, N3, N4), data = NMA_template_infection_without, sm = "RR")

NMA_infections_netmeta <- netmeta(TE, seTE, treat1, treat2, studlab, data = NMA_infections, comb.fixed = FALSE, comb.random = TRUE)

NMA_infections_sum <- summary(NMA_infections_netmeta)

NMA_infections_sum

#Pscore

NMArank <- netrank(NMA_infections_netmeta, small.values = "bad")

#Forest NMA infections

forest(NMA_infections_netmeta, reference.group="Placebo", sortvar = c("Placebo", 'CBD 10 mg/kg', 'CBD 20 mg/kg'), drop.reference.group=F, rightcols=c("effect", "ci", "Pscore"), rightlabs="P-Score", small.values="bad",col.inside = "blue", col.study = "blue", col.diamond = "green", xlim = c(0.1, 5), text.addline1 = "\n\n\n\n Heterogenity: I^2 = 71%, tau^2=0.5634, p = 0.02", ff.addline = 3)

#efficacy: less than 50 percent seizures

efficacy_50less <- metabin(Ee, Ne, Ec, Nc, data = EXCEL_FOR_R_efficacy_event_50_percent_less_seizures_both_dosages, studlab = paste(author), comb.fixed = FALSE, comb.random = TRUE, method.tau = "SJ",hakn = FALSE, incr = 0.1, sm = "RR")

summary(efficacy_50less)

#forest efficacy: less than 50 percent seizures

forest(efficacy_50less, col.inside = "blue", col.study = "blue", col.diamond = "green", xlim = c(0.1, 10))

#convulsions

convulsions.bin <- metabin(Ee, Ne, Ec,Nc, data = EXCEL_FOR_R_convulsions, studlab = paste(author), comb.fixed = FALSE,comb.random = TRUE, method.tau = "SJ", hakn = FALSE, incr = 0.1, sm = "RR")

summary(convulsions.bin)

#forest convulsions:

forest(convulsions.bin, col.inside = "blue", col.study = "blue", col.diamond = "green", xlim = c(0.01, 20))

convulsions_CBD.bin <- metabin(Ee, Ne, Ec,Nc, data = EXCEL_FOR_R_convulsions_CBD, studlab = paste(author), comb.fixed = FALSE,comb.random = TRUE, method.tau = "SJ", hakn = FALSE, incr = 0.1, sm = "RR")

forest(convulsions_CBD.bin, col.inside = "blue", col.study = "blue", col.diamond = "green", xlim = c(0.01, 20))

convulsions_with_THC.bin <- metabin(Ee, Ne, Ec,Nc, data = EXCEL_FOR_R_convulsions_with_THC, studlab = paste(author), comb.fixed = FALSE,comb.random = TRUE, method.tau = "SJ", hakn = FALSE, incr = 0.1, sm = "RR")

forest(convulsions_with_THC.bin, col.inside = "blue", col.study = "blue", col.diamond = "green", xlim = c(0.01, 20))

CGIC metamedian:

library(metamedian)

#Treatment – Devinsky 2017

A1 = c(7, rep(6, 4), rep(5,3), rep(4,15), rep(3,18), rep(2,10), rep(1,9))

#Placebo – Devinsky 2017

A2 = c(6, rep(5, 6),rep(4,31), rep(3, 12), rep(2, 4), rep(1, 4))

#Treatment –Miller 2020

B1 = c(7, rep(6,3), rep(5,5), rep(4,17), rep(3,19), rep(2,10), rep(1,11))

#Placebo –Miller 2020

B2 = c(rep(6,2), rep(5,4), rep(4,32), rep(3,18), rep(2,8), rep(1,1))

max.Treat <- c(max(A1), max(B1))

max.placebo <- c(max(A2), max(B2))

mean.Treat <- c(mean(A1), mean(B1))

mean.placebo <- c(mean(A2), mean(B2))

med.Treat <- c(median(A1), median(B1))

med.placebo <- c(median(A2), median(B2))

min.Treat <- c(min(A1), min(B1))

min.plaecbo <- c(min(A2), min(B2))

n.Treat <- c(length(A1), length(B1))

n.placebo <- c(length(A2), length(B2))

quantile(A1)

#output 0% 25% 50% 75% 100%

#output 1 2 3 4 7

quantile(B1)

#output 0% 25% 50% 75% 100%

#output 1 2 3 4 7

q1.Treat <- c(2, 2)

quantile(A2)

#output 0% 25% 50% 75% 100%

#output 1 3 4 4 6

quantile(B2)

#output 0% 25% 50% 75% 100%

#output 1 3 4 4 6

q1.placebo <- c(3, 3)

q3.Treat <- c(4,4)

q3.placebo <- c(4,4)

metamedian::qe(min.g1 = min.Treat, q1.g1 = q1.Treat, med.g1 = med.Treat, q3.g1 = q3.Treat, max.g1 = max.Treat, n.g1 = n.Treat, min.g2 = min.plaecbo, q1.g2 = q1.placebo, med.g2 = med.placebo, q3.g2 = q3.placebo, max.g2 = max.placebo, n.g2 = n.placebo)

#output Random-Effects Model (k = 2; tau^2 estimator: REML)

#output tau^2 (estimated amount of total heterogeneity): 0 (SE = 0.1138)

#output tau (square root of estimated tau^2 value): 0

#output I^2 (total heterogeneity / total variability): 0.00%

#output H^2 (total variability / sampling variability): 1.00

#outputTest for Heterogeneity:

#outputQ(df = 1) = 0.0000, p-val = 1.0000

#outputModel Results:

#output estimate se zval pval ci.lb ci.ub

#output -1.0000 0.2001 -4.9977 <.0001 -1.3922 -0.6078 ***

#output Signif. codes: 0 ‘***’ 0.001 ‘**’ 0.01 ‘*’ 0.05 ‘.’ 0.1 ‘ ’ 1

#Forest plot of CGIC

A = metamedian::qe(min.g1 = min.Treat, q1.g1 = q1.Treat, med.g1 = med.Treat, q3.g1 = q3.Treat, max.g1 = max.Treat, n.g1 = n.Treat, min.g2 = min.plaecbo, q1.g2 = q1.placebo, med.g2 = med.placebo, q3.g2 = q3.placebo, max.g2 = max.placebo, n.g2 = n.placebo)

forest(A, slab=c("Devinsky et al. 2017", "Miller et al. 2020"), showweights=TRUE, refline = NA, xlab = "Median CGIC under 20 mg/Kg CBD in comparison to placebo", col = "green")

text(-2.7, -3.5, paste('Heterogeneity: I² = 0%, τ²=0.000, p <0.001'), cex = 0.9, xpd=NA)

#Box plot of median results:

boxplot(A1,A2,B1,B2, at=c(1,2.5,5,6.5), range = 1.5, col = c("darkgreen","blue","darkgreen", "blue"), border = c("black", "black", "brown", "brown"), names = c("20mg/kg", "Placebo", "20mg/kg", "Placebo"))

text(c(0.5,9), -1, paste('***P-value<0.001'), xpd=NA)

text(5,7.1, "***", col = "red")

text(1,7.1, "***", col = "black")

**Supplementary Table S2: Summary findings table**

| **Measured outcome** | **Figure and panel** | **Number of studies included in the analysis** | **Number of patients in the analysis** | **Effect size, 95%CI** | **Heterogeneity measured in *I^2^*** |
| --- | --- | --- | --- | --- | --- |
| 50% reduction in seizures in CBD treatment vs. Placebo | 1A | 2 | 316 | 1.69 [1:20 – 2.36] | 0% |
| Reported seizures events in medical cannabinoids vs. Placebo | 1B | 5 | 707 | 0.71 [0.41 – 1.24] | 23% |
| Reported seizures events in CBD products vs. Placebo | 1C | 3 | 353 | 0.59 [0.36 – 0.96] | 14% |
| Reported seizures events in CBD:THC mixed products vs. Placebo | 1D | 2 | 354 | 1.35 [0.48 – 4.03] | 0% |
| CGIC median measurement | 2A | 2 | 316 | -1 [-1.39 – (-0.61)] | 0% |
| Serious adverse events in medical cannabinoids treatment | 3A | 6 | 745 | 1.63 [0.80 – 3.29] | 0% |
| Decreased appetite events in CBD treatment vs. Placebo | 4A | 5 | 642 | 2.10 [0.96 – 4.62] | 22% |
| Network Meta-analysis of decreased appetite events in 2 doses CBD treatment vs. Placebo | 4B | 5 | 632 | CBD 5mg/kg: 0.32 [0.02 – 4.24]  CBD 10mg/kg: 1.23 [0.61 – 2.47]  CBD 20mg/kg: 2.40 [1.39 – 4.15] | 0% |
| Gastrointesitnal hyperactivity events in medical cannabinoids treatment vs. Placebo | 4C | 5 | 706 | 1.55 [0.92 – 2.61] | 62% |
| Gastrointesitnal hyperactivity events in CBD treatment vs. Placebo | 4D | 3 | 352 | 2.30 [1.28, 4.12] | 0% |
| Gastrointesitnal hyperactivity events in CBD:THC mixed products vs. Placebo | 4E | 2 | 354 | 1.04 [0.63, 1.73] | 0% |
| Mean difference of vomits and retching in Nabilone vs control | S3 | 2 | 48 | -11.5 [-17.9 – (-5.1)] | 0% |
| Events of infections under Medical Cannabinoids treatment vs. Placebo | S4A | 4 | 424 | 0.91 [0.47 – 1.77] | 75% |
| infections in 2 doses CBD treatment vs. Placebo | S4B | 3 | 342 | CBD 5mg/kg: 1.14 [0.27 – 4.91]  CBD 10mg/kg: 0.72 [0.22 – 2.38]  CBD 20mg/kg: 1.05 [0.38– 2.82] | 71% |
| Pyrexia events in Medical Cannabinoids vs. Placebo | S4C | 4 | 424 | 1.41 [0.60 – 3.33] | 0% |

**Supplementary Figure S1: search algorithm diagram**

*two articles contained mixed data collected from adult patients while one article was a brief report, which currently did not yield any data for pooled analysis and will be fully published in the future; One additional article was substantially different from other in its study design and its quality, and therefore did not meet inclusion criteria

**Supplementary Figure S2: Quality assessment of the included studies according to RoB-2 scale**


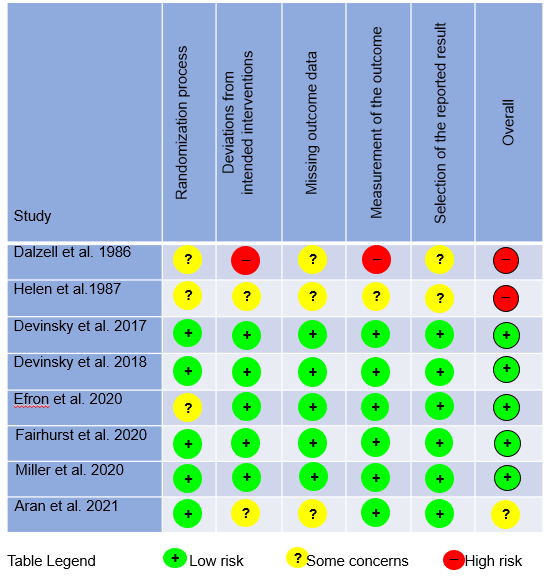


**Supplementary Figure S3: Efficacy of nabilone versus conventional control as an antiemetic treatment**

Meta-analysis of mean difference of vomits and retching in Nabilone vs control (p-value<0.0001). This analysis inlcude 48 patients.

**Supplementary Figure S4a-c: events of infections and pyrexia under medical cannabinoids treatment**

**a)**
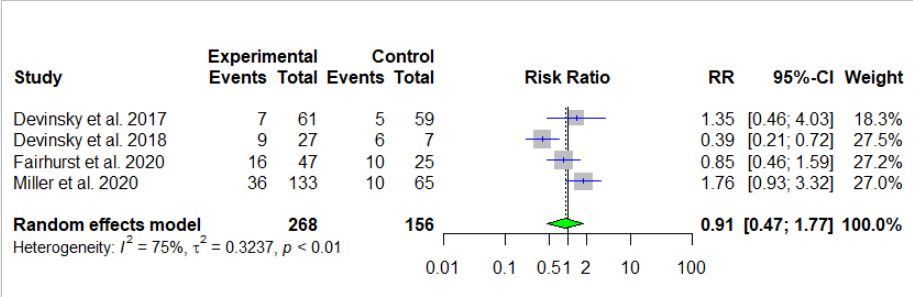


**b)**


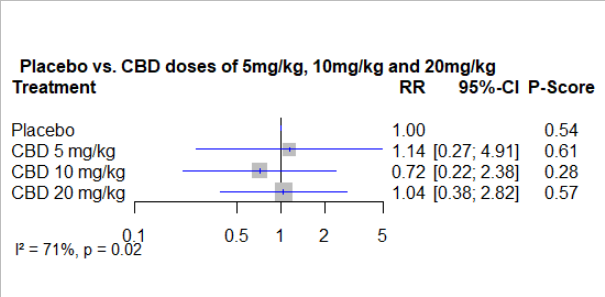


**c)**
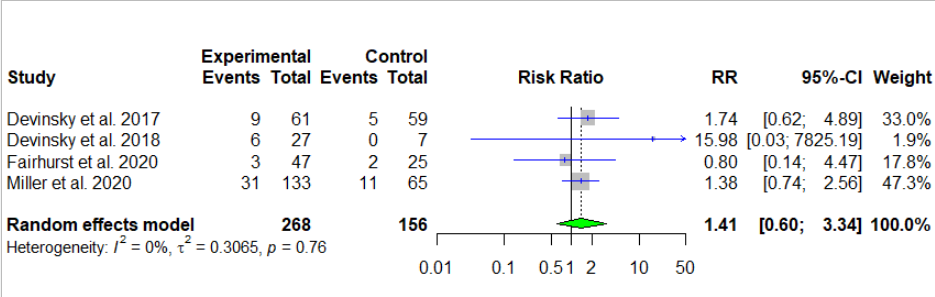


a) Meta-analysis of events of infections under Medical Cannabinoids treatment vs. Placebo (p-value=0.78).

b) Network Meta-analysis of infections in 3 doses CBD treatment vs. Placebo.

c) Meta-analysis of pyrexia events in Medical Cannabinoids vs. Placebo (p-value=0.43).
